# Synthesis of Silver Nanoparticles Using Aqueous Extract of Medicinal Plants' (*Impatiens balsamina* and *Lantana camara*) Fresh Leaves and Analysis of Antimicrobial Activity

**DOI:** 10.1155/2019/8642303

**Published:** 2019-07-01

**Authors:** Henry F. Aritonang, Harry Koleangan, Audy D. Wuntu

**Affiliations:** Physical Chemistry Division, Faculty of Mathematics and Natural Sciences, Sam Ratulangi University, Jalan Kampus Unsrat Kleak, Manado 95115, Indonesia

## Abstract

Plant-mediated synthesis of nanomaterials has been increasingly gaining popularity due to its eco-friendly nature and cost-effectiveness. In the present study, we synthesized silver (Ag) nanoparticles using aqueous extracts of fresh leaves of *Impatiens balsamina* and *Lantana camara* medicinal plants as bioreducing agents. This method allowed the synthesis of nanoparticles, which was confirmed by ultraviolet-visible (UV-Vis) spectrophotometry and transmission electron microscopy (TEM). UV-Vis spectra and visual observation showed that the color of the fresh leaf extracts of *L. camara* and *I. balsamina* turned into grayish brown and brownish yellow, respectively, after treatment with Ag precursors. In addition, TEM analysis confirmed that AgNO_3_ solutions for all concentrations produced Ag nanoparticles and their average size was less than 24 nm. Moreover, aqueous leaf extracts of *I. balsamina* and *L. camara* were separately tested for their antimicrobial activity against Gram-positive *Staphylococcus aureus* and Gram-negative *Escherichia coli* bacteria. The results showed that the bacterial growth was inhibited by the extracts containing Ag nanoparticles. Statistical calculation performed using the Tukey test showed that zones of inhibition for the two bacteria produced by the aqueous leaf extracts of *L. camara* containing 3 mM and 5 mM Ag precursors were not significantly different from that by ciprofloxacin as positive control. On the contrary, there was significant difference between the zone of inhibition for *E. coli* by ciprofloxacin and that by the extracts of *I. balsamina* leaves containing 3 mM and 5 mM Ag precursors. A similar result was observed on the zone of inhibition for *S. aureus* by the extracts of *I. balsamina* leaves containing 3 mM Ag precursor. It was shown that the aqueous extracts of fresh *L. camara* leaves containing Ag nanoparticles were comparable to ciprofloxacin in inhibiting bacterial growth.

## 1. Introduction

Nanoparticles represent a particle with a nanometer size of 1–100 nm. The nanoscale material has new, unique, and superior physical and chemical properties compared to its bulk structure, due to an increase in the ratio of the surface area per volume of the material/particle [1]. The most widely studied nanoparticle materials are metal nanoparticles because they are easier to synthesize. Moreover, these materials have a wide range of applications: detectors, catalysts, surface coating agents, and antibacterial/antimicrobials, among many others. Some of the most studied metallic nanoparticles include silver (Ag) [2, 3], gold (Au) [4], platinum (Pt) [5–7], and palladium (Pd) [8].

Ag nanoparticle is an interesting metal to be studied, especially in the field of health and medicine. Ag is a strong antibacterial and also toxic to cells. Ag has the ability to damage bacterial cell walls, inhibits bacterial cell growth, and disrupts cell metabolism because of the interaction between Ag ions with macromolecules in cells, such as proteins and deoxyribonucleic acid (DNA). The ion Ag that interacts with the cell prevents protein synthesis, further decreases the membrane permeability, and eventually leads to cell death. The Ag nanoparticles are chemically more reactive than Ag in their bulk. Therefore, Ag nanoparticles are indicated to have stronger antibacterial capabilities [9–11].

Ag nanoparticles can be synthesized through several methods, including chemical reduction. Chemical reduction methods are often used because they are easier and economical [12]. This method is done by reducing Ag salts by reducing agents, such as sodium citrate or sodium borohydride [13]. However, the use of chemicals in the synthesis of Ag nanoparticles results in the adsorption of toxic chemicals (reducing agents and organic solvents) on the surface of the material so that it will have adverse and harmful effects on its application [14]. Therefore, the use of environmentally friendly methods is desirable.

Green synthesis methods for synthesizing nanoparticles using natural products can be used to address the problem by utilizing plants or microorganisms [15]. The utilization of plants in the biosynthesis of nanoparticles involves the content of secondary metabolites as reducing agents [16]. Allegedly, biological agents act as reducers, stabilizers, or both in the process of forming nanoparticles [17]. The biosynthesis of Ag nanoparticles has been carried out by utilizing a number of plants and evaluating the antimicrobial activity, such as ethanol extracts from *Cardiospermum halicacabum* L. leaves [18], *Impatiens balsamina* L. leaves [19], and *Lantana camara* L. fruits [20]. The ethanol extract of *I. balsamina* and *L. camara* was obtained from the leaves which were first dried for further analysis of its antimicrobial activity.

*I. balsamina* and *L. camara* are one of the most common medicinal plants in Indonesia, especially for wound dressing [19, 21]. According to Meenu et al. [22], the dry *I. balsamina* ethanol extract contains chemical compounds such as essential oils, phenols, flavonoids, carbohydrates, proteins, alkaloids, glycosides, iridoid glycosides, phenylethanoids, oligosaccharides, quinine, saponins, steroids, triterpenoids, sesquiterpenoids, and tannins and has antibacterial activity against *Staphylococcus epidermidis*. The aqueous extract of fresh leaves of *I. balsamina* and *L. camara* to synthesize Ag nanoparticles (extract Ag nanoparticles) is important to be investigated mainly because it can be used as a wound medicine that can be packaged in the form of infusions/fluids. Liquids that use water solvents are much safer for health and the environment than using chemical solvents [23]. Therefore, in this study, we used water as a solvent in medicinal plant extracts.

The present study synthesized Ag nanoparticles using aqueous extracts of fresh leaves of *I. balsamina* and *L. camara* and then evaluated its antimicrobial activity, particularly against the growth of *Staphylococcus aureus* (*S. aureus*) and *Escherichia coli* (*E. coli*) bacteria.

## 2. Materials and Methods

### 2.1. Chemicals and Plant Material Collection

**Figure 1:**
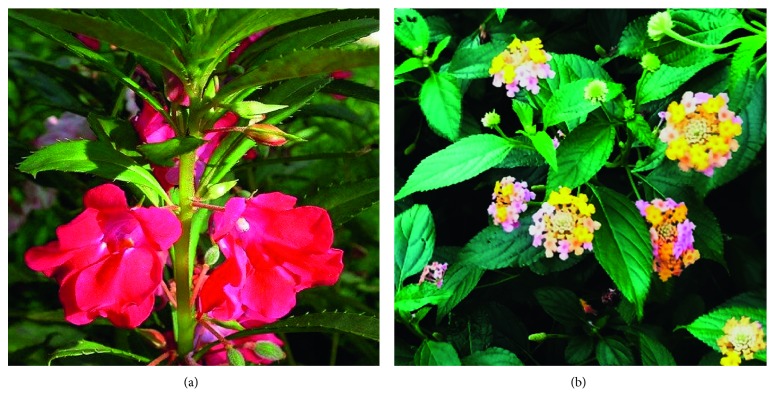
Plants: (a) *Impatiens balsamina*; (b) *Lantana camara*.

All the reagents purchased were of analytical grade and used without any further purification. Silver nitrate (AgNO_3_) was purchased from Sigma-Aldrich with a ≥99.5% purity. Fresh leaves of *I. balsamina* and *L. camara* were collected from the surroundings of the Bitung region, North Sulawesi, Indonesia (Figure 1). Distilled water was used for preparing aqueous solutions all over the experiments.

### 2.2. Preparation of Leaf Extract

Aqueous leave extracts were prepared by the following procedure: fresh leaves of *I. balsamina* were collected and washed with tap water at first, and then the surface was washed under running water with distilled water until no impurities remained. Then, the fresh leaves were cut into small pieces, and 10 g was weighed and put into a beaker with 100 ml of distilled water. The mixture was heated for 20 minutes at 60°C while stirring occasionally and then allowed to cool at room temperature [24]. The mixture was filtered using the Whatman 42 filter paper and then centrifuged at 81 G-force for 20 minutes. The extract was stored in the refrigerator for further use to synthesize Ag nanoparticles from AgNO_3_ precursor solution. The same process was also done on fresh leaves of *L. camara*.

### 2.3. Synthesis of Ag Nanoparticles

AgNO_3_ powder was dissolved in distilled water to prepare 10 mM AgNO_3_ stock solution from which a series of 1 mM, 2 mM, 3 mM, 4 mM, and 5 mM AgNO_3_ solutions were prepared. The AgNO_3_ solutions were mixed with the aqueous extract of *I. balsamina* fresh leaves at a ratio of 1 : 1 (v/v) to a volume of 50 mL in a flask. The flask was wrapped with an aluminum foil and was then heated in a waterbath at 60°C for 5 hours. Furthermore, the mixture was stored in the refrigerator for the antibacterial activity test and further analyzed by using UV-Vis spectrophotometer and TEM. The same procedure was also carried out on the aqueous extract of *L. camara* fresh leaves.

### 2.4. Assay for Antimicrobial Activity of Ag Nanoparticles against Microorganisms

All equipment and growing media were sterilized by autoclaving at 115°C and 15 psi for 30 minutes. The antimicrobial activity has been investigated against *S. aureus* as a model for Gram-positive bacteria and *E. coli* as a model for Gram-negative bacteria. The antimicrobial activity was evaluated by the disc diffusion method. Preparation of the bacteria stock was done to reproduce and rejuvenate bacteria. This was done by inoculating each one inoculation loop pure culture of *E. coli* and *S. aureus* into 5 ml of nutrient agar solution and then incubated at 37°C for 24 hours in the incubator. Preparation of test bacteria was carried out by inserting one inoculation loop of cultured bacteria into 5 ml of 0.19% NaCl solution. Furthermore, it was vortexed and its turbidity was adjusted to 0.5 McFarland solution turbidity of ∼10^8^ CFU/mL by adding the cultured bacteria [19].

The inhibition method was used in evaluating the antibacterial activity. 20 ml of nutrient agar solution was put into a Petri dish, sterilized for 15 minutes until the nutrient agar solution became solid, and then 0.1 ml of bacterial solution was applied to the nutrient agar growing medium. Thereafter, negative control (distilled water), positive control (Ciprofloxacin), and sample (2 replications) were placed. Next, it was incubated at 37°C for 24 hours before the clear zone diameter was measured using the sliding term. To see the ability of each leaf extract containing Ag nanoparticles in inhibiting bacterial growth compared to ciprofloxacin positive control, a statistical analysis was performed using the Tukey test at the 95% confidence level.

### 2.5. Characterization of Ag Nanoparticles

The reduction of pure Ag^+^ ions was monitored by measuring the UV-Vis spectrum of the reaction medium after diluting a small aliquot of the sample into distilled water. The color change in the reaction mixture (metal ion solution + *I. balsamina* extract) was recorded through visual observation. UV-Vis spectral analysis was done by using UV-Vis spectrophotometer UV-1800 (Shimadzu) at the wavelength of 200–800 nm. JEOL JEM-1400 Transmission Electron Microscope (TEM), operating at 120 V and an acceleration voltage of 15 kV, was used to analyze the morphology and size of Ag nanoparticles. For TEM measurements, extract samples containing Ag nanoparticles were dispersed on a copper grid and dried at room temperature. The particle sizes of the Ag nanoparticles were measured using Image J software. The histogram of the size distribution was established by Origin software.

## 3. Results and Discussion

### 3.1. UV-Vis Spectra Analysis

**Figure 2:**
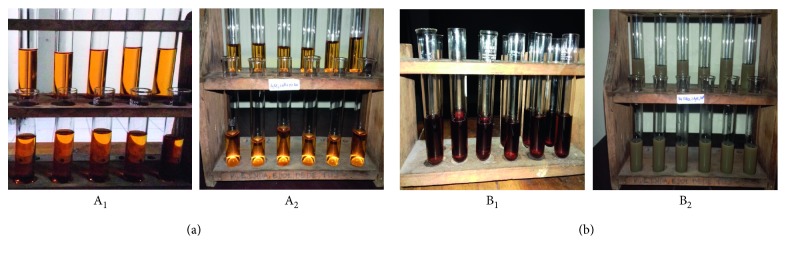
Aqueous extract from fresh leaves of (a) *I. balsamina* and (b) *L. camara*. Before (A_1_ and B_1_) and after (A_2_ and B_2_) the addition of AgNO_3_ solution.

The aqueous extract of fresh leaves of *I. balsamina* and *L. camara* change their colors when warmed. The *I. balsamina* extract changes color from colorless to brownish yellow, while *L. camara* becomes yellowish brown (Figure 2).

This warm extract solution changed color again after adding AgNO_3_ solution. Color changes are possible because some of the Ag ions begin to be reduced due to the effects of heat and produces Ag^+^ complex. This complex was responsible for changing color from brownish yellow to grayish brown (*L. camara*), while the *I. balsamina* extract remained a brownish yellow (Figure 2 (A_2_ and B_2_)). This color change indicates the formation of Ag nanoparticles [25].

**Figure 3:**
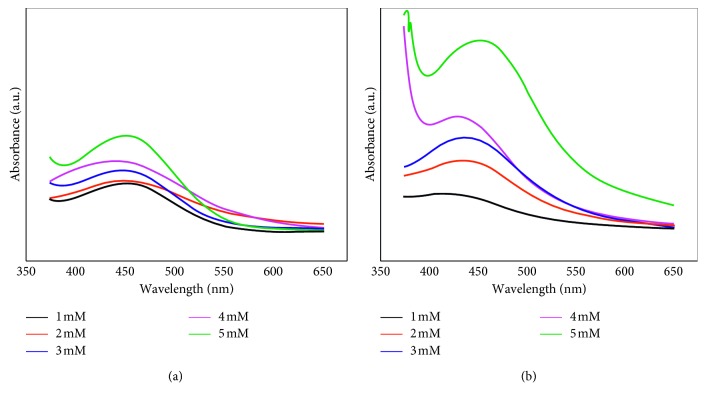
UV-Vis absorbance spectrum of Ag nanoparticles as a function of AgNO_3_ concentration in aqueous extracts of fresh leaves of (a) *I. balsamina* and (b) *L. camara*, respectively.

The Ag nanoparticles synthesized in each extract solution was analyzed using UV-Vis spectroscopy. This was done to determine the characteristics of the peak spectrum of the Ag nanoparticle wavelength prepared for each different AgNO_3_ concentrations (1 mM–5 mM) (Figure 3).

The characteristics of Ag nanoparticles normally appear at a wavelength interval of 400–600 nm [26]. UV-Vis spectra of Ag nanoparticles synthesized using the *I. balsamina* aqueous extract evince the blue shift of the absorption band with increasing AgNO_3_ concentration. For 1 mM, 2 mM, 3 mM, 4 mM, and 5 mM samples, the absorption peak is centered around 450–420 nm. This information shows that the Ag nanoparticles have formed in the extract, where the Ag^+^ has been reduced to Ag^0^. Proteins and all secondary metabolites of extract play a critical role in both the reducing and capping mechanism for nanoparticle formation [25]. The Ag nanoparticles contained in the aqueous extract of the *L. camara* also exhibit similar characteristics, where the shift of the absorption band with increasing AgNO_3_ concentrations. However, the shift of the absorption peak was a little narrower than that of the Ag nanoparticles synthesized with the *I. balsamina* aqueous extract, where the absorption peak is centered on 450–440 nm. The peak wavelength of Ag nanoparticles in aqueous fresh leaf extracts can be seen in Table 1.

### 3.2. TEM Analysis

**Figure 4:**
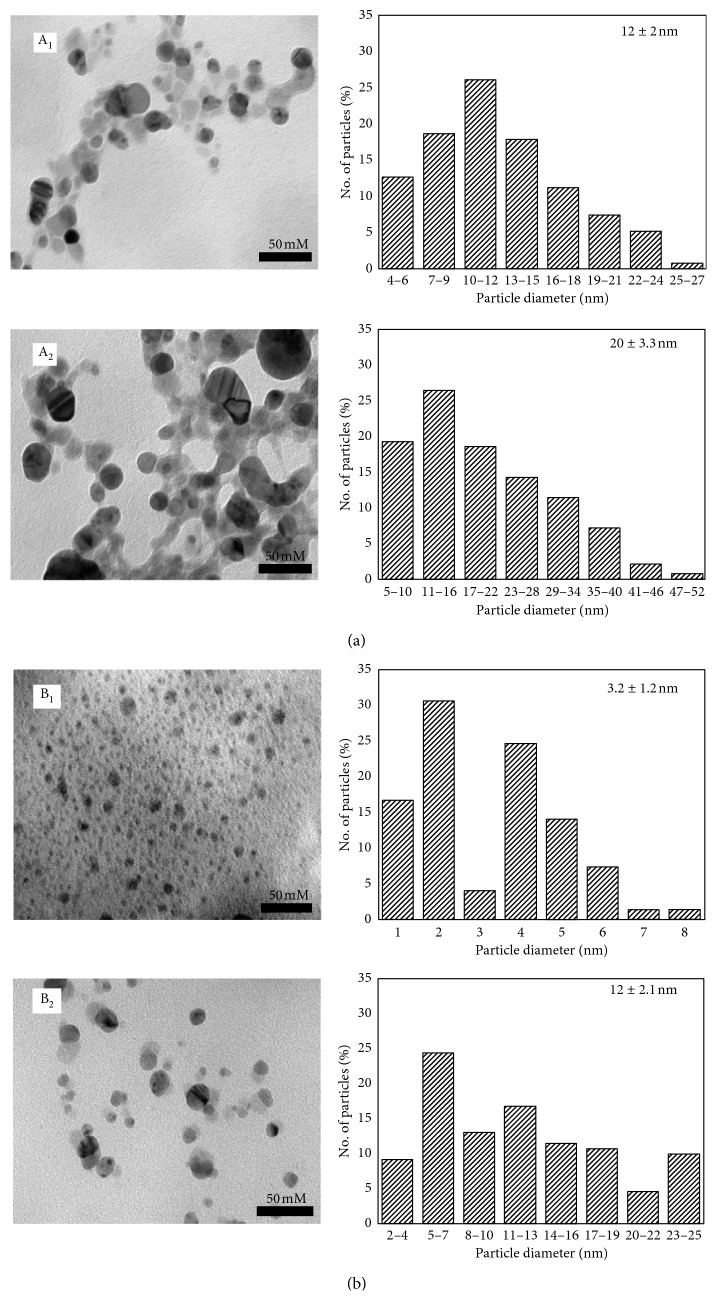
TEM images of Ag nanoparticles in the aqueous extract of (a) *I. balsamina* and (b) *L. camara* and their particle-size distribution at different molar concentrations of AgNO_3_: 1 mM (A_1_ and B_1_) and 5 mM (A_2_ and B_2_).

The size and morphology of Ag nanoparticles synthesized using aqueous extracts of fresh leaves have been evaluated by TEM analysis. The obtained TEM images of the Ag nanoparticles prepared by each leaf extract from *I. balsamina* and *L. camara* are shown in Figure 4.

The nanoparticles are quite polydispersed and a layer of the organic material surrounding the synthesized Ag nanoparticles could explain the good dispersion of these nanoparticles in solution. Generally, the Ag nanoparticles synthesized using aqueous extracts are well dispersed although some of them were noted to be agglomerated. Notably, the majority of the particles in the TEM images are not in physical contact with each other but appeared separated by the organic layer. Therefore, TEM images clearly indicate the coating of Ag nanoparticles with an organic layer. The presence of several polyphenolic components including flavonoids and terpenoids facilitated the reduction of Ag ions and also stabilized the surface of the resultant Ag nanoparticles [25].

The Ag ions quantity influenced the size of the particles. When AgNO_3_ concentration is increased to 5 mM, an obvious change in the size distribution of nanospheres was observed (Table 2).

### 3.3. Antibacterial Activity Studies

**Figure 5:**
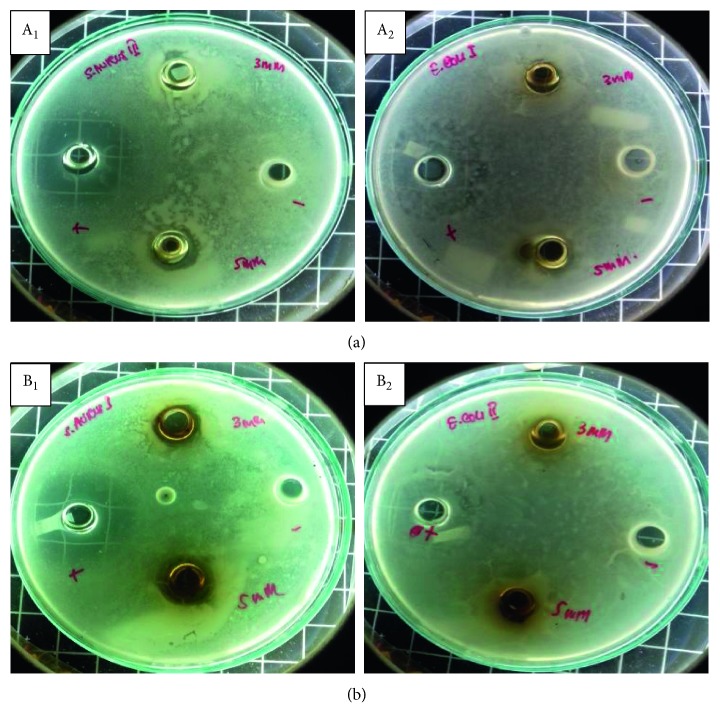
Antibacterial activity of Ag nanoparticles synthesized using aqueous leaf extracts of *I.balsamina* (a) and *L. camara* (b) against Gram-positive *S. aureus* (A_1_ and B_1_) and Gram-negative *E. coli* bacteria (A_2_ and B_2_).

The present study revealed that the tested leaf extracts of *I. balsamina* and *L. camara* medicinal plants showed potent antibacterial activity against two bacterial strains: Gram-positive *S. aureus* and Gram-negative *E. coli*. Aqueous extracts of *I. balsamina* and *L. camara* containing Ag nanoparticles showed activity in all Ag concentrations tested against all bacteria (Figure 5).

Antibacterial activity was shown by an inhibition zone which was characterized by a clear zone between the wells (containing samples) and a certain distance. Formation of inhibition zones around the wells shows bacterial sensitivity to antibacterial and antibiotic ingredients (which are used as positive controls). The positive control used in the well was a ciprofloxacin 500 mg solution and functioned as a control of the test solution by comparing the diameter of the inhibition zone formed. On the contrary, distilled water as negative control was used to determine the effect of solvents in the test solution on the growth of *S. aureus* and *E. coli* bacteria. It was clear that it was the extracts containing Ag nanoparticles that had the antibacterial activity, not the solvent.

The diameter of inhibition zones formed for each concentration of the AgNO_3_ precursor added to the aqueous extracts of fresh leaves of *I. balsamina* and *L. camara* medicinal plants in synthesizing Ag nanoparticles is presented in Table 3.

Table 3 shows that the antibacterial activity against *S. aureus* was increased, which was indicated by an increase in the inhibition zone diameter from 11.03 mm to 13.8 mm, with the increasing Ag concentration in *I. balsamina* extract. However, the opposite result was shown by *E. coli*, which was from 10.2 mm to 8.9 mm. The same was true for Ag nanoparticles in *L. camara* aqueous extracts which were from 13.9 mm to 15.8 mm and 17.7 mm to 15.4 mm for *S. aureus* and *E. coli*, respectively. While the distilled water negative control did not show inhibition zone for all treatments, ciprofloxacin positive control showed similar inhibition zone diameter, which average above 19 mm, for the two bacteria with the increasing Ag concentration in leaves extracts of the two plants.

However, based on the results of statistical analysis, it was shown that only three treatments were significantly different than the positive controls. The variables were aqueous extracts of *I. balsamina* leaves containing Ag nanoparticles (3 mM) against *S. aureus* and *E. coli* bacteria and aqueous extracts of *I. balsamina* leaves containing Ag nanoparticles (5 mM) against *E. coli* bacteria.

This information was supported by data that the average size of Ag nanoparticles synthesized using *L. camara* extract was relatively smaller than that using the extract of *I. balsamina*. The results of this study were also supported by previous studies that the small size of Ag nanoparticles makes these particles easier to penetrate the outer wall of bacteria, enter the body, destroy the respiratory chain, and thus inhibit cell respiration, causing bacterial death [27, 28]. Regarding the inhibition zone, the antibacterial activity of Ag nanoparticles synthesized in this study was categorized into strong inhibitory activity (inhibition zone of 10–19 mm) according to Davis and Stout [29].

## 4. Conclusions

Medicinal plants, namely, aqueous extracts of fresh leaves of *I. balsamina* and *L. camara*, can be used as bioreduction agents to produce Ag nanoparticles. The formation of Ag nanoparticles in the extract was observed by the color change of *I. balsamina* extract into brownish yellow while of *L. camara* extract into grayish brown. Color changes that occur indicate that Ag particles have formed. The Ag particles produced had an increased size due to the increased concentration of AgNO_3_ solution, but the average size is still in nanometer. Ag nanoparticles contained in the extract were able to inhibit the growth of *S. aureus* and *E. coli* bacteria, and the best antibacterial activity was exhibited by the *L. camara* extract containing Ag nanoparticles.

## Figures and Tables

**Table 1:** Peak wavelength and absorbance of Ag nanoparticles in aqueous extracts of fresh leaves of *I. balsamina* and *L. camara*.

Concentration (mM)	Wavelength (nm)	Absorbance
*I. balsamina*		
1	452	0.894
2	452	0.937
3	451	1.109
4	441	1.285
5	455	1.736

*L. camara*		
1	420	0.667
2	438	1.277
3	438	1.691
4	433	2.059
5	450	3.386

**Table 2:** Size of Ag nanoparticles produced from various concentrations of AgNO_3_ using aqueous extracts of fresh leaves of *I. balsamina* and *L. camara*.

Concentration of AgNO_3_ solution (mM)	Ag nanoparticle size (nm)	
	*I. balsamina*	*L. camara*
1	12 ± 2	3.2 ± 1.2
2	15 ± 2.1	4 ± 1
3	17 ± 2.2	6 ± 1.1
4	19 ± 2.5	10 ± 1.3
5	20 ± 3.3	12 ± 2.1

**Table 3:** Antibacterial activity of Ag nanoparticles synthesized using various concentrations of AgNO_3_ precursors and aqueous extracts of fresh leaves of *I. balsamina* and *L. camara*.

Plants	[AgNO_3_] (mM)/replication		Zone of inhibition (mm)			
			*S. aureus*	Positive control	*E. coli*	Positive control
*I. balsamina*	3	1	11.3	18.2	14.3	17.8
		2	12.3	21.2	7.8	20.7
		3	9.5	19.7	8.5	20
	Average		*11.03* ^a^	*19.7* ^b^	*10.2* ^c^	*19.5* ^d^
	5	1	20	19.7	10.3	19.6
		2	10.5	19.8	7.3	19.4
		3	11	19.6	9	19.5
	Average		**13.8**	**19.7**	*8.9* ^e^	*19.5* ^f^

*L. camara*	3	1	12.8	18.1	20.5	18
		2	14.5	20.9	15.3	21
		3	14.5	20	17.3	19.8
	Average		**13.9**	**19.7**	**17.7**	**19.6**
	5	1	13	19.6	15.3	19.4
		2	20	19.8	16.5	19.7
		3	14.3	19.8	14.5	19.8
	Average		**15.8**	**19.73**	**15.4**	**19.63**

Positive control = ciprofloxacin; values indicated with “a” are significantly different from values indicated with “b”; values indicated with “c” are significantly different from values indicated with “d”; values indicated with “e” are significantly different from values indicated with “f”.

## Data Availability

The data used to support the findings of this study are available from the corresponding author upon request.
